# Supplementary material for: Intranasal Inoculation with Classical Swine Fever Virus Provided a More Consistent Experimental Disease Model Compared to Oral Inoculation
Source: Vet Sci. 2024 Jan 28;11(2):56. doi: 10.3390/vetsci11020056 (PMC10892780; doi:10.3390/vetsci11020056)
Supplement: Supplementary file 1 [file vetsci-11-00056-s001.zip › vetsci-2775884-supplementary.pdf]

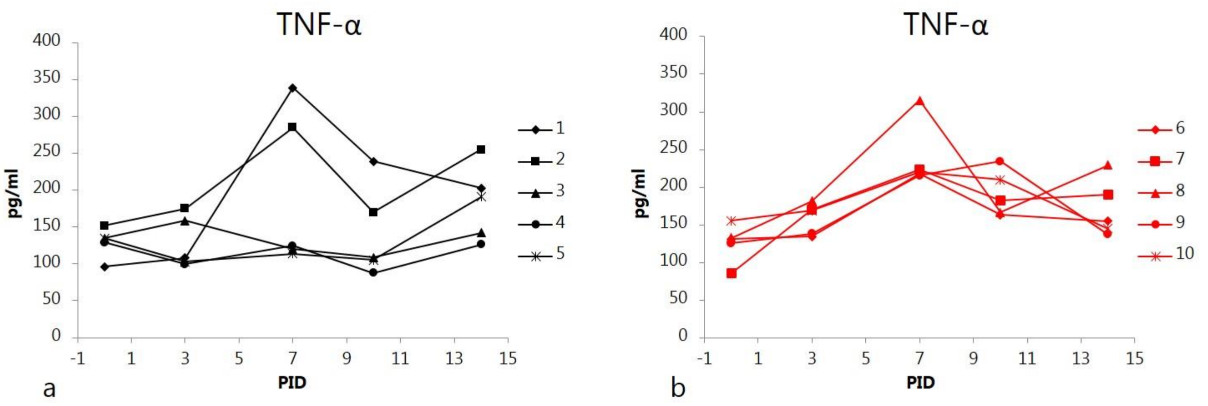

Supplementary Figure S1. Variations over time in TNF-*α* concentration in the peripheral blood of individual pigs orally (ORAL group; a) or intranasally (NASAL group; b) inoculated with classical swine fever virus strain Paderborn. PID = post-inoculation day.

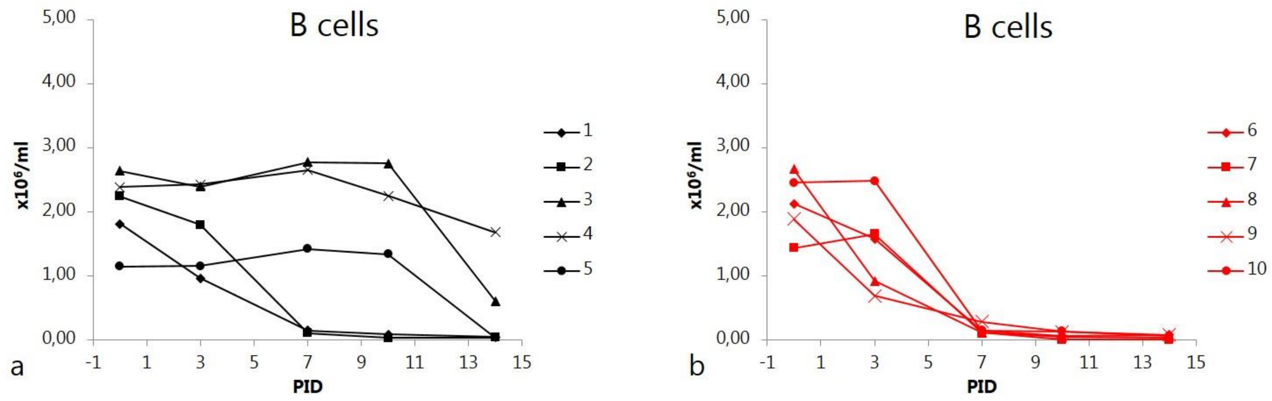

Supplementary Figure S2. Variations over time in absolute numbers of B cells in the peripheral blood of individual pigs orally (ORAL group; a) or intranasally (NASAL group; b) inoculated with classical swine fever virus strain Paderborn. PID = post-inoculation day.

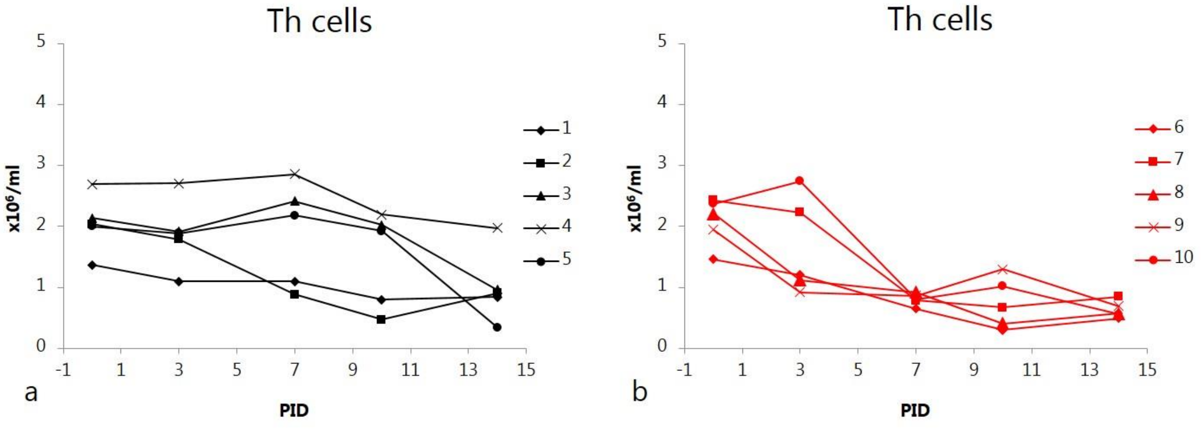

Supplementary Figure S3. Variations over time in absolute numbers of T helper (Th) cells in the peripheral blood of individual pigs orally (ORAL group; a) or intranasally (NASAL group; b) inoculated with classical swine fever virus strain Paderborn. PID = post-inoculation day.

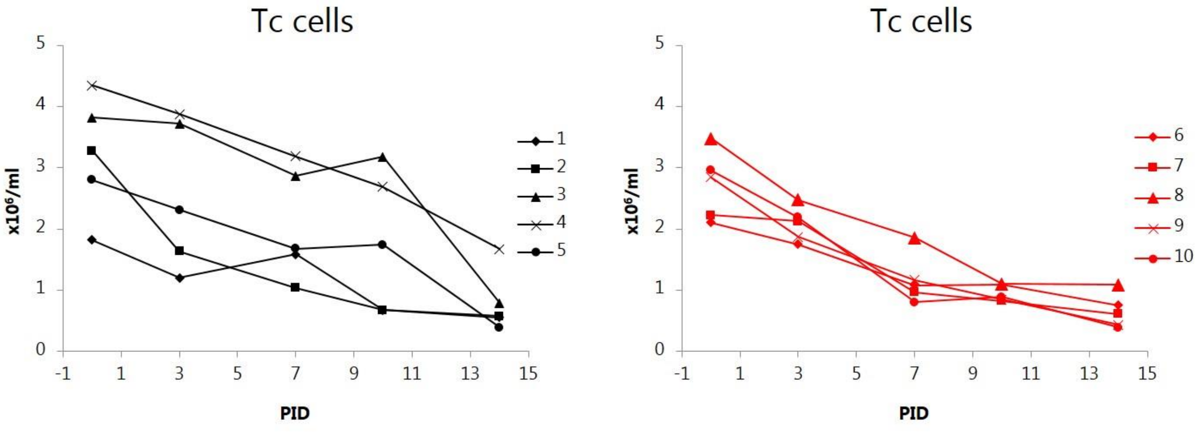

Supplementary Figure S4. Variations over time in absolute numbers of cytotoxic T (Tc) cells in the peripheral blood of individual pigs orally (ORAL group; a) or intranasally (NASAL group; b) inoculated with classical swine fever virus strain Paderborn. PID = post-inoculation day.
